# Measurement invariance of the satisfaction with leisure satisfaction scale by gender, marital status, and age

**DOI:** 10.1186/s41155-023-00282-y

**Published:** 2024-01-02

**Authors:** Elif Köse, Hüseyin Gökçe, Neşe Toktaş, Tennur Yerlisu Lapa, Evren Tercan Kaas

**Affiliations:** 1 Department of Recreation, Faculty of Sports Sciences, Akdeniz University, Antalya, Turkey; 2 Department of Sport Management, Faculty of Sports Sciences, Pamukkale University, Denizli, Turkey; 3 Department of Sports and Health Sciences, Faculty of Sports Sciences, Akdeniz University, Antalya, Turkey; 4 Department of Sport Management, Faculty of Sports Sciences, Akdeniz University, Antalya, Turkey

**Keywords:** Leisure satisfaction, Measurement invariance, Gender, Marital status, Age

## Abstract

**Background:**

Leisure satisfaction is the degree of positive perception and emotions that an individual acquires as a result of participating in leisure time activities, and it has an important function in maintaining and increasing leisure time participation. Some studies on leisure satisfaction address the comparisons between groups. These studies are based on the premise that the measurement tool used to reveal the between-group differences measures the same feature in subgroups.

**Objective:**

In this study, we investigated whether the differences between the groups were due to the measurement tool by examining the psychometric properties of the leisure satisfaction scale.

**Methods:**

The study sample comprised 2344 exercising individuals, including 1228 (52.3%) women and 1116 (47.6%) men. The structural invariance of the leisure satisfaction scale, developed by Beard and Ragheb (Journal of Leisure Research 12:20-33, 1980) and adapted into Turkish by Gökçe and Orhan (Spor Bilimleri Dergisi 22:139–145, 2011), was tested through multiple-group confirmatory factor analysis.

**Results:**

The results indicated that the structural and metric invariance conditions were fulfilled across gender, marital status, and age in all subscales of the leisure satisfaction scale. Scalar invariance was obtained in educational and social satisfaction subscales across gender and in physical satisfaction subscale across marital status.

**Conclusion:**

The study provides evidence for the future comparisons according to these three variables, indicating that the differences obtained will result from the real differences between groups rather than the measurement tool properties.

## Introduction

Leisure activities refer to activities outside of work, in which individuals can participate by choosing them freely (Beard & Ragheb, 1980). Although people seem to be very lucky in terms of technology and luxury today, the situation is a little different. People have difficulties in dealing with many physical, social, and spiritual problems during their daily lives (Gulam, 2016). At this point, recreation stands at the point of great need for people to overcome these problems. Leisure satisfaction (LS) is defined as positive perceptions and emotions that an individual reveals, obtains, and reaches as a result of participating in leisure activities, indicating the degree of satisfaction of the individual (Beard & Ragheb, 1980). As the level of LS increases, participation in leisure activities also increases, and the individual’s life satisfaction is positively affected (Beard & Ragheb, 1980; Losier et al., 1993).

According to Iso-Ahola and Weissinger (1990), among the psycho-social factors that affect LS, there are various components that make up the structure of leisure time. These are participation, gender, leisure time attitudes, beliefs, leisure motivation, frequency of activity, leisure ethics, and values. Similarly, LS may vary depending on activity characteristics. Beard and Ragheb (1980) expressed this in leisure satisfaction scale (LSS) with six different sub-dimensions: educational, physical, physiological, social, relaxation, and aesthetic sub-dimensions.

Leisure activities may have different characteristics. Among leisure activities involving cultural, social, and physical activities offer various benefits (Bum et al., 2018). Ho (2008) summarizes benefits obtained from leisure activities as follows: Physical Benefits such as healthy development of the body, disease prevention and control, energy regeneration, body fat control; psychologic benefits such as decrease in depression and anxiety, increase in self-confidence and self-esteem; social benefits such as strengthening family relations and social bonds; educational benefits such as development of inspiring talents and acquisition of new knowledge; economic benefits such as providing new employment opportunities and increase in an individual’s income; environmental benefits such as increasing the environmental consciousness; relaxation benefits such as coping with daily stress and positively changing mood; aesthetical benefits such as meeting the spiritual needs; emotional benefits such as life satisfaction and happiness. When the literature is analyzed, it can be observed that studies mentioning physical and psychological benefits of leisure activity participation attract considerable attention from researchers. Participation in leisure activities has numerous positive effects on the physical health (e.g., prevention of chronic conditions, including cardiovascular diseases, some cancers, type 2 diabetes, hypertension, and non-communicable diseases like obesity and all-cause mortality risks; Booth et al., 2012; Langhammer et al., 2018). It also positively influences psychological parameters such as psychological well-being (Bum et al., 2018), life satisfaction, happiness (An et al., 2020), quality of life, LS, and life satisfaction (Tokay Argan & Mersin, 2021).

Sustainable participation in leisure activities is important for a healthy life. In general, it is known that high satisfaction from leisure activities has a more positive effect on sustainable participation compared to other factors, such as participation motivation, service quality, and perceived value (Bum et al., 2018). It is emphasized that there is a strong relationship between LS and participation, and individuals who get a high level of satisfaction from leisure activities tend to participate more in activities and feel less depression and anxiety (Pressman et al., 2009). Also the psychological well-being of these individuals are reported to be higher (Lee et al., 2022).

The above-mentioned requirements emerging in fields such as health, sports, and tourism have allowed for the transformation of leisure and recreation into a huge industry. As an 8.8 trillion-dollar industry, it is one of the largest industries in the world (Australian Leisure Management, 2019). With regard to the industry, increasing or maintaining profitability is highly related to the satisfaction and loyalty of people who have experience (Helgesen, 2006). LS is quite important for understanding the satisfaction of visitors and updating services. Also the concept of *leisure satisfaction*, which is one of the predictors of leisure participation, still maintains its popularity in the field since 1980.

Therefore, the precision and accuracy of the results obtained when measuring LS are very important for both the academic field and this emerging industry. In the process of adapting this scale developed to measure LS into our language, the removal of inequalities related to translation and the elimination of language-related differences within the scope of expert opinions may not guarantee that the scale has the same meaning in two different cultures or that it is understood equally between groups (Brislin, 1980). Therefore, the results obtained from studies addressing differences between groups may differ depending on the demographic characteristics of individuals and may also be due to the bias of the measurement tool (Başusta & Gelbal, 2015). In this case, the results obtained from studies, in which the invariance of the measurement tool is not tested, are questionable. In other words, the differences between groups obtained for a measurement tool, in which measurement invariance is not tested, cannot considered a “real” difference (Mark & Wan, 2005). In view of the literature, it is observed that the interest in measurement invariance studies that test the construct validity of measurement tools and reveal that the measurement tool does not work biasedly has increased (Casanova et al., 2021; Lima-Castro et al., 2021). Previous studies on the construct validity of LS investigated the measurement invariance of the Life Satisfaction Scale (LSS) according to the gender variable (Ahn & Song, 2021). In addition to Ahn and Song’s (2021) study, in this study, we aimed to test the measurement invariance of the LSS developed by Beard and Ragheb (1980) according to not only gender but also age and marital status variables. In this respect, we believe that our study fills an important gap in the field and will provide important empirical evidence to researchers using this measurement tool.

## Method

### Participants and procedure

Fifty-eight thousand and seven hundred fifty adult individuals registered to sports centers in Antalya province constituted the study population. Within the scope of the study, 2928 individuals were accessed, with a response rate of 93.1% (*n* = 2726) However, he study was conducted with 2344 individuals after data screening. The participants, consisting of women (Mean age = 35.10 ± 12.42) by 52.3% (*n* = 1228) and men (Mean age = 29.71 ± 13.13) by 47.6% (*n* = 1116), were 17–80 years old. Of these participants, 45.13% (*n* = 1058) were married and 54.8% (*n* = 1286) were single. While 20.2% (*n* = 474) of individuals doing regular physical activity were 17–21 years old, 21.6% (*n* = 506) were 22–25 years old, 26.5% (*n* = 621) were 26–36 years old, and 31.7% (*n* = 743) were 37 years old or older. The convenience sampling method, one of non-probability sampling methods, was employed as a sampling method. Generalizations are made as a result of the studies conducted with sampling techniques with known probability in social sciences. However, although the non-probability sampling method was employed in this study, it was aimed to increase the reliability and validity of the related research results by reaching a large observation set (Faber & Fonseca, 2014). Graduate-level students were assigned to each sports facility in order to collect the data. Adult individuals who did physical activity on a regular basis were asked whether they would like to evaluate the output of their recreational physical activity. Volunteers who wanted to participate were asked to fill in the ethical consent form and detailed information about the purpose of the research was provided to them both verbally and in writing. Furthermore, the participants were informed that they can withdraw from the study at any time and that the personal information obtained would remain confidential. Participants were allocated 10 minutes to answer the questions, and they were asked to respond to each question completely. Institutional permission from the Akdeniz University Clinical Research Ethics Committee was obtained to conduct the study (KAEK-539).

### Data collection tools

LSS was developed by Beard and Ragheb (1980), reorganized as a short form by Idyll Arbor, Inc. in 2002, and adapted into Turkish by Gökçe and Orhan (2011). LSS is a measurement tool that aims to reveal the satisfaction of individuals with leisure activities. It is a measurement tool consisting of 6 factors, including psychological, educational, social, physical, relaxation, and aesthetic factors, and 24 items. The scale items are listed and scored as (1) “Almost never important” and (5) “Almost always true.” It was determined that Cronbach’s alpha reliability coefficients of the sub-dimensions of the scale ranged from 0.76 to 0.80, and the total reliability coefficient of the scale was .90.

### Data analysis

In the current study, SPSS package program was used to obtain the descriptive statistics and test the basic assumptions. In order to provide evidence for the structural validity, measurement invariance according to genders, marital status, and age was studied. Multiple-group confirmatory factor analysis (MG-CFA), considered to be one of the structural equation modelling methods, was used to test the invariance between genders, marital status, and age. During the derivation of the statistics, the maximum likelihood estimation method was preferred. This estimation method was used since the data were in a form of continuous variable. The quality of the data is very important in multivariate statistical practices such as confirmatory factor analysis (CFA) and MG-CFA (Mertler et al., 2021). To improve the quality of the study, all the basic assumptions related to the analysis (i.e., missing and extreme values, size and normality of the data set, and multicollinearity) were tested. The first of these basic assumptions involves identifying the extreme values. Univariate and multivariate (mahlonobis distance) extreme values and missing data (*n* = 382) were excluded from the original data set consisting of 2726 people.

It was determined that the missing data is below 1% and is not systematically distributed. To test the normality assumption, the arithmetic mean, mode, median, kurtosis, and skewness coefficients of the data were evaluated (Table 1).

**Table 1:** Descriptive statistic for LSS

Items	Mean (SD)	Median	Mode	Skewness	Kurtosis	α
Item1	3.504 (1.151)	4.00	4.00	−.485	−.445	.824
Item 2	4.001 (.921)	4.00	4.00	−.904	.712	
Item 3	4.014 (.924)	4.00	4.00	−.887	.619	
Item 4	3.899 (1.016)	4.00	4.00	−.721	−.079	
Item 5	3.921 (.943)	4.00	4.00	−.687	.014	.838
Item 6	3.979 (.944)	4.00	4.00	−.757	.069	
Item 7	4.098 (.895)	4.00	4.00	−.969	.783	
Item 8	3.933 (.941)	4.00	4.00	−.745	.242	
Item 9	4.036 (.908)	4.00	4.00	−.913	.748	.846
Item 10	3.933 (.922)	4.00	4.00	−.678	.164	
Item 11	3.690 (.963)	4.00	4.00	−.417	−.259	
Item 12	3.884 (.946)	4.00	4.00	−.610	−.093	
Item 13	4.230 (.800)	4.00	5.00	−.848	.188	.857
Item 14	4.256 (.790)	4.00	5.00	−.899	.329	
Item 15	4.161 (.843)	4.00	5.00	−.799	.166	
Item 16	4.092 (.876)	4.00	4.00	−.731	.066	
Item 17	3.352 (1.165)	3.00	3.00	−.309	−.659	.783
Item 18	3.786 (.969)	4.00	4.00	−.479	−.314	
Item 19	3.896 (.908)	4.00	4.00	−.526	−.208	
Item 20	4.126 (.840)	4.00	4.00	−.704	−.162	
Item 21	4.079 (.876)	4.00	4.00	−.782	.222	.877
Item 22	3.891 (.931)	4.00	4.00	−.620	.016	
Item 23	4.006 (.888)	4.00	4.00	−.813	.556	
Item 24	3.822 (.961)	4.00	4.00	−.521	−.216	

For the item discrimination analysis carried out within the scope of the internal consistency criterion, a t-test was performed in independent groups, and it was determined that each item was 27% discriminatory for upper and lower groups (Table 2).

**Table 2:** t-Test results for item discrimination

Items	Bottom 27% Top 27%	*N*	Mean	Std. Deviation	*df*	t-value	*p*
Item1	Bottom 27% Top 27%	632	2.6669 4.2848	.93617 .95934	1262	−30.343	0.000
Item 2	Bottom 27% Top 27%	632	3.1440 4.6946	.89363 .54302	1262	−37.280	0.000
Item 3	Bottom 27% Top 27%	632	3.1424 4.7563	.93800 .46506	1262	−38.753	0.000
Item 4	Bottom 27% Top 27%	632	2.9794 4.7185	.94603 .52165	1262	−40.470	0.000
Item 5	Bottom 27% Top 27%	632	3.1155 4.6566	.89952 .59647	1262	−35.897	0.000
Item 6	Bottom 27% Top 27%	632	3.1458 4.7437	.93946 .48508	1262	−37.992	0.000
Item 7	Bottom 27% Top 27%	632	3.2676 4.7753	.94035 .43988	1262	−36.512	0.000
Item 8	Bottom 27% Top 27%	632	3.1187 4.6013	.93539 .63386	1262	−32.986	0.000
Item 9	Bottom 27% Top 27%	632	3.2184 4.6775	.95199 .52803	1262	−33.696	0.000
Item 10	Bottom 27% Top 27%	632	3.1392 4.6171	.91280 .57598	1262	−34.422	0.000
Item 11	Bottom 27% Top 27%	632	2.9396 4.3861	.90374 .72514	1262	−31.384	0.000
Item 12	Bottom 27% Top 27%	632	3.0285 4.6013	.89326 .57898	1262	−37.144	0.000
Item 13	Bottom 27% Top 27%	632	3.5253 4.8054	.83276 .41191	1262	−34.637	0.000
Item 14	Bottom 27% Top 27%	632	3.5728 4.7848	.82036 .45865	1262	−32.420	0.000
Item 15	Bottom 27% Top 27%	632	3.4509 4.7563	.85054 .50430	1262	−33.188	0.000
Item 16	Bottom 27% Top 27%	632	3.3481 4.7215	.84769 .52961	1262	−34.543	0.000
Item 17	Bottom 27% Top 27%	632	2.6630 4.0174	.98097 1.09226	1262	−23.193	0.000
Item 18	Bottom 27% Top 27%	632	3.0095 4.5380	.82928 .72873	1262	−34.807	0.000
Item 19	Bottom 27% Top 27%	632	3.1282 4.6203	.79282 .64050	1262	−36.803	0.000
Item 20	Bottom 27% Top 27%	632	3.4335 4.7220	.82089 .51376	1262	−33.449	0.000
Item 21	Bottom 27% Top 27%	632	3.3608 4.6946	.88994 .54593	1262	−32.118	0.000
Item 22	Bottom 27% Top 27%	632	3.0712 4.6044	.90412 .58387	1262	−35.814	0.000
Item 23	Bottom 27% Top 27%	632	3.2231 4.6820	.89508 .52674	1262	−35.313	0.000
Item 24	Bottom 27% Top 27%	632	3.0380 4.5424	.91396 .68157	1262	−33.172	0.000

Scatter plots were also examined to evaluate the multivariate normality and linearity distribution conditions. It has been determined that the scatter plots are elliptical and are suitable for multivariate normality and linearity conditions (Tabachnick et al., 2007). When evaluated in the context of the size of the data set, it can be stated that the research sample is quite good. The multicollinearity problem is another assumption of multivariate statistics. Variance Inflation Factor (VIF) and Condition Index (CI) indices were examined to determine whether the relevant condition was met (Oribe-Garcia et al., 2015). The VIF value is usually less than 10 and the CI value is less than 30, indicating that there is no multicollinearity problem (VIF < 2.5; CI < .30).

After testing the study’s basic assumptions, CFA analysis was performed in the second step. In this study, we analysed chi-square (χ^2^), degree of freedom (df), comparative fix index (CFI), Normed fit index (NFI), non-normed fit index (NNFI), root mean square error of approximation (RMSEA), and standardized root mean square residual (SRMR) fit indices (Table 3).

**Table 3:** Baseline CFA models for LS

Group	χ^2^	*df*	χ^2^/*df*	*p* < 0,01	CFI	NFI	NNFI	RMSEA (90% CI)	SRMR
Gender									
Women	1991.58	237	8.40	.000	.98	.97	.97	.078 (.075–.081)	.053
Men	1718.24	237	7.24	.000	.97	.97	.97	.075 (.072–.078)	.056
Marital Status									
Single	1578.61	237	6.60	.000	.98	.98	.98	.066 (.063–.070)	.050
Married	2414.13	237	10.18	.000	.97	.97	.97	.093 (.090–.097)	.064
Age									
17–21	778.44	237	3.28	.000	.98	.97	.97	.069 (.063–.075)	.055
22–25	781.47	237	3.29	.000	.98	.97	.97	.067 (.062–.073)	.052
26–36	909.29	237	3.83	.000	.98	.97	.98	.068 (.063–.072)	.051
37+	1961.33	237	8.27	.000	.97	.96	.96	.099 (.095–.010)	.073
Total	3419.79	237	14.42	.000	.97	.97	.98	.076 (.073–.078)	.053

Table 3 presents the results of the CFA analysis by gender, age, and marital status. χ^2^/*df* indicated moderate fit (≤5) by age (17–21, 22–25, 26–36), while χ^2^/*df* obtained for other variables (women-men, married-single, and over 37 years old) indicated poor fit. The large sample size affects χ^2^/*df* results (Tabachnick et al., 2007). Hence, it is important to evaluate other indices less affected by the sample size (Brown, 2015). Upon examining RMSEA values, it was revealed that the fit index obtained for married people and individuals over 37 years of age indicated moderate fit (.08 ≤ RMSEA ≤ .10; Kelloway, 1998), while the RMSEA value (≤.08) indicated good fit in other groups (Hooper et al., 2008). Upon evaluating other fit indices for CFA analysis, the CFI, NFI, NNFI, and SRMR values showed good and perfect fit (Marsh et al., 2006). The fact that more than one fit index indicates perfect fit criteria for all subgroups can be interpreted as the validation of the model. As a result of CFA analysis, factor loadings were determined to be ≥ .40 in all subgroups.

**Fig. 1:**
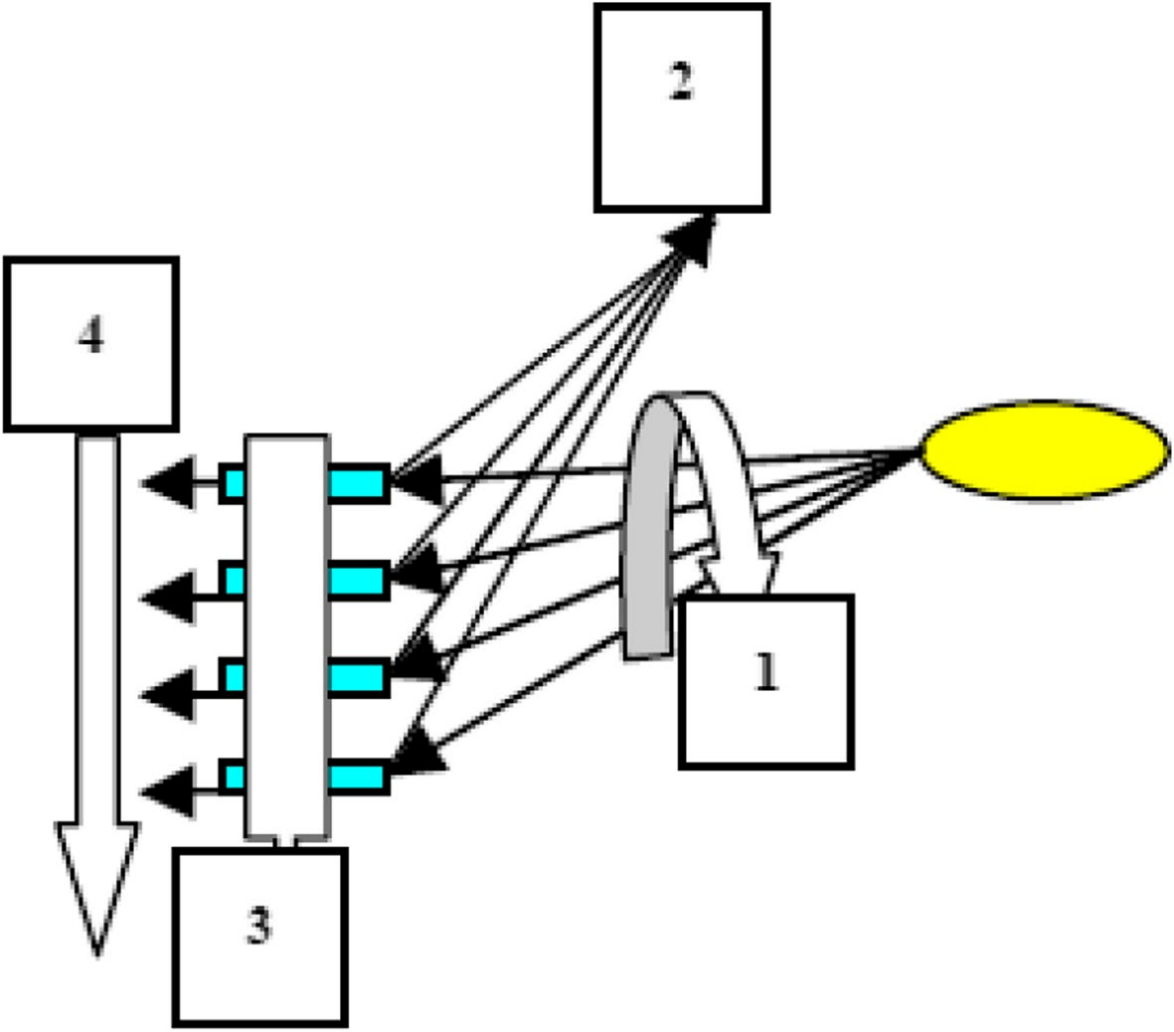
Measurement invariance hierarchy (Başusta, 2010)

The third step of the study consists of testing the measurement invariance. Before the MG-CFA analysis, the analysis was carried out in light of 8 guiding principles in the COSMIN checklist Cross-cultural validity\measurement invariance (Prinsen et al., 2018). Measurement invariance is a statistical test that allows application with all kinds of data except experimental methods. It is a type of covariance analysis and is designed to test a particular construct in different groups. When the studies using measurement invariance are examined, it is seen that the most commonly used method is MG-CFA. Measurement invariance is a four-stage process (Fig. 1). It starts from the model in which no constraints are introduced, and the equivalence of parameters between groups is examined up to the most limited model, and this process continues gradually (Horn et al., 1983).

#### Structural invariance

Structural invariance is the first stage of measurement invariance. At this stage, no restrictions are imposed on the model (Horn et al., 1983). Structural invariance is based on the hypothesis that the structure of the model is the same in groups. In cases where structural invariance is satisfied, it is inferred that the measurement tool measures the same psychological structure for subgroups (Bollen, 1989).

#### Metric invariance

Metric invariance is the second stage after structural invariance is achieved. Metric invariance is based on the hypothesis that subgroups perceive scale items in the same way (Steenkap & Baumgartner, 1998). If metric invariance cannot be achieved between groups, it is indicated that there are differences between the item scores observed for these groups, and it is not meaningful to make comparisons between groups (Başusta, 2010).

#### Scalar invariance

Scalar invariance is the third stage of measurement invariance. Achieving scalar invariance at this stage is based on the hypothesis that differences between groups based on items do not change with context (Chen et al., 2005). Achieving scalar invariance indicates that item tendencies and item constants are equal between groups (Tucker et al., 2006).

#### Strict invariance

Strict invariance is the last stage of measurement invariance and is based on the hypothesis that the error terms, in other words, the specific variances of the items in a measurement tool, are invariant between groups.

There are different views in the literature on which fit indices should be evaluated at the reporting stage of measurement invariance. Some researchers consider reporting only ΔCFI sufficient since the ΔCFI value is the most appropriate fit index in explaining the relationship between the latent and observed scores, while other researchers suggest that ∆RMSEA and ∆SRMR values should be reported together with the ΔCFI value at the scalar invariance stage (Chen, 2007; Cheung & Rensvold, 2002). In this study, ΔCFI (Wu et al., 2007), ∆RMSEA, and ∆SRMR (Chen, 2007; Cheung & Rensvold, 2002) values were examined instead of ∆χ^2^, which is a strict criterion (Cheung & Rensvold, 2002). The cut-off points for ∆CFI, ∆RMSEA, and ∆SRMR values are determined according to the sample size. In cases where the sample size is greater than 300, the criteria to be taken are considered as ≤.010 for ∆CFI, ≤.015 for ∆RMSEA, and ≤ .030 for ∆SRMR (Chen, 2007).

## Results

Table 4 shows the factor loadings of all subgroups. The factor loadings range from .53 to .85 for women, from .38 to .68 for men, from .36 to .66 for single individuals, from .51 to .94 for those married individuals, from .36 to .77 for 17–21 years old; from .50 to .76 for 22–25 years old, from .43 to .73 for 26–36 years old, from .40 to .80 for 37+ years old, and from .50 to .88 for all groups.

**Table 4:** Standardized factor loadings for all groups

Item	Women Std factor loading/ t value	Man Std factor loading/ t value	Single Std factor loading/ t value	Married Std factor loading/ t value	17–21 Std factor loading/ t value	22–25 Std factor loading/ t value	26–36 Std factor loading/ t value	37+ Std factor loading/ t value	All Std factor loading/ t value
I1	.75/24.02	.43/17.42	.44/18.18	.77/23.70	.50/11.41	.50/11.46	.52/12.66	.58/18.68	.66/19.41
I2	.80/29.02	.66/28.24	.63/25.03	.88/28.19	.75/18.59	.71/18.17	.67/18.59	.74/23.19	.83/26.88
I3	.85/33.19	.68/28.94	.64/30.61	.94/30.42	.73/18.62	.72/19.71	.73/21.47	.80/23.99	.87/28.58
I4	.82/29.27	.60/26.83	.59/29.93	.88/26.02	.69/18.27	.65/18.15	.68/20.20	.76/20.49	.73/22.28
I5	.79/28.32	.57/23.47	.57/25.11	.83/26.98	.69/15.75	.64/15.98	.66/17.98	.67/21.23	.76/23.53
I6	.82/31.08	.61/24.79	.61/28.45	.86/27.04	.66/17.69	.72/18.23	.71/19.93	.72/21.03	.70/20.72
I7	.82/31.52	.64/25.71	.66/29.66	.82/27.39	.77/19.22	.76/18.81	.74/20.05	.62/20.37	.74/22.49
I8	.80/29.44	.55/22.20	.58/25.04	.81/26.52	.69/16.07	.65/15.57	.68/18.23	.63/20.33	.74/22.65
I9	.79/29.69	.63/25.82	.61/26.79	.84/28.21	.69/15.77	.72/17.62	.72/20.72	.65/20.84	.75/22.67
I10	.77/28.21	.60/24.43	.58/25.99	.82/25.91	.66/15.27	.64/15.97	.73/20.56	.64/19.47	.78/24.02
I11	.75/25.95	.50/20.07	.52/21.94	.75/23.68	.60/13.26	.55/13.41	.63/16.98	.60/18.55	.74/22.29
I12	.80/28.02	.56/23.22	.56/25.11	.82/25.57	.62/15.21	.62/15.26	.67/18.33	.68/20.55	.76/23.35
I13	.74/28.21	.63/24.98	.63/27.58	.79/25.68	.75/16.81	.72/17.68	.72/20.25	.57/18.59	.82/26.15
I14	.74/28.34	.61/23.33	.60/25.67	.78/25.75	.68/15.61	.67/15.87	.72/19.81	.59/19.01	.88/29.04
I15	.78/26.01	.57/22.93	.56/25.38	.76/23.75	.68/15.19	.64/15.26	.65/18.50	.60/18.61	.79/24.59
I16	.78/25.91	.58/23.42	.56/24.88	.76/24.25	.71/16.01	.60/14.90	.62/17.04	.68/19.87	.62/17.83
I17	.53/16.28	.38/10.89	.36/12.83	.51/13.86	.36/8.60	.58/6.67	.43/11.51	.40/10.37	.50/13.47
I18	.70/24.12	.49/18.74	.51/21.57	.67/20.56	.58/12.84	.54/13.43	.60/16.63	.54/15.64	.81/24.64
I19	.74/25.02	.54/21.01	.53/24.77	.70/22.88	.69/15.66	.61/15.05	.67/17.03	.57/17.84	.86/26.74
I20	.72/26.33	.56/21.68	.59/24.37	.71/22.93	.74/16.20	.61/14.69	.64/17.09	.55/17.87	.61/17.13
I21	.69/26.02	.57/21.09	.58/24.83	.72/23.07	.74/16.95	.64/14.02	.60/17.06	.56/17.81	.70/21.08
I22	.79/29.01	.57/22.30	.58/25.18	.88/26.79	.71/16.41	.59/14.38	.68/18.30	.66/21.04	.84/26.91
I23	.79/30.22	.59/22.92	.60/25.96	.83/27.36	.73/16.44	.64/16.01	.70/19.10	.64/20.97	.88/27.52
I24	.77/27.32	.49/19.60	.53/22.42	.78/24.93	.62/14.18	.62/12.43	.66/18.02	.64/19.44	.74/22.60

Table 5 shows the results of measurement invariance for the sub-dimensions of LS according to gender. Considering the structural invariance, which is the first step of measurement invariance, it was observed that the fit indices for *χ*^2^/*df* values in all sub-dimensions were above the expected cut-off value, and RMSEA values were also high in all sub-dimensions, except for the Relaxation sub-dimension. Although the NNFI value was slightly lower in sociological and aesthetic sub-dimensions, other fit indices (NFI, CFI, and SRMR) indicated a perfect fit. Upon examining the NFI, NNFI, CFI, and SRMR values obtained for the Psychological, Educational, Physical, and Relaxation sub-dimensions, it was determined that the obtained fit indices indicated a perfect fit. The perfect fit in more than one fit index indicates that structural invariance is satisfied for all sub-dimensions.

**Table 5:** Fit statistics of measurement invariance stages by gender

	Gender	*χ*^*2*^	*df*	*χ*^*2*^*/df*	NFI	NNFI	CFI	SRMR	RMSEA (90% CI)	∆CFI	∆SRMR	∆RMSEA	Decision
Psychological	Structural	81.9	4	20.47	.98	.95	.98	.025	.129(.11–.15)	–	–	–	H_0_ Accept
	Metric	85.93	8	10.74	.98	.97	.98	.033	.091(.074–.011)	0	.008	−.038	H_0_ Accept
	Scalar	169.63	15	11.03	.96	.97	.96	.046	.94(.064–.100)	−.02	.021	−.811	H_0_ Reject
Educational	Structural	75.86	4	18.96	.98	.95	.98	.026	.124(.10–.15)	–	–	–	H_0_ Accept
	Metric	82.82	8	10.35	.98	.97	.98	.036	.089(.073–.11)	0	.010	−.035	H_0_ Accept
	Scalar	146.20	15	9.74	.97	.98	.97	.031	.086(.074–.099)	−.01	.005	−.038	H_0_ Accept
	Strict	203.95	19	10.73	.95	.97	.96	.056	.091(.080–.10)	−.02	.030	−.033	H_0_ Reject
Social	Structural	195.91	4	48.97	.96	.88	.96	.049	.202(.18–.23)	–	–	–	H_0_ Accept
	Metric	220.61	8	27.57	.96	.94	.96	.054	.151(.13–.17)	0	.005	−.051	H_0_ Accept
	Scalar	287.66	15	19.17	.94	.96	.95	.070	.125(.11–.14)	.01	.021	−.077	H_0_ Accept
	Strict	317.81	19	16.72	.94	.96	.94	.079	.116(.10–.13)	.02	.030	−.086	H_0_ Reject
Physical	Structural	166.68	8	20.83	.97	.96	.97	.047	.130(.11–.15)	–	–	–	H_0_ Accept
	Metric	199.89	12	16.65	.96	.97	.97	.10	.116(.10–.13)	0	.053	−.014	H_0_ Accept
	Scalar	338.64	19	18.82	.94	.96	.94	.12	.120(.11–.13)	−.03	.073	−.010	H_0_ Reject
Aesthetic	Structural	195.47	4	48.86	.95	.85	.95	.051	.202(.18–.23)	–	–	–	H_0_ Accept
	Metric	199.35	8	24.91	.95	.93	.95	.056	.143(.13–.16)	0	.005	−.059	H_0_ Accept
	Scalar	596.84	19	31.41	.84	.90	.84	.15	.161(.15–.17)	−.11	.099	−.041	H_0_ Reject
Relaxation	Structural	61.14	8	7.64	.99	.99	.99	.029	.075(.058–.093)	–	–	–	H_0_ Accept
	Metric	92.99	12	7.74	.98	.99	.99	.089	.076(.062–.091)	0	.060	.001	H_0_ Accept
	Scalar	303.67	19	15.98	.94	.97	.95	.10	.113(.10–.12)	−.04	.071	.038	H_0_ Reject

The CFI, RMSEA, and SRMR values obtained in metric invariance were compared with the CFI, RMSEA, and SRMR values obtained from the structural model. As seen in Table 4, it was determined that the ∆RMSEA value obtained for the psychological, educational, sociological, and aesthetic sub-dimensions was above the expected cut-off value (∆RMSEA ≥ .015), but the ∆CFI (∆CFI ≤ .010) and ∆SRMR values (∆SRMR ≤.030) were below the expected cut-off value. In the Physical and Relaxation sub-dimensions, it was found that the ∆SRMR value (∆SRMR ≥.030) was above the expected cut-off value, but the ∆CFI (∆CFI ≤ .010) and ∆RMSEA values (∆RMSEA ≤ .015) were below the expected cut-off value. The fact that more than one fit index meets the expected criteria indicates that metric invariance is satisfied in all sub-dimensions. The fit indices obtained for the scalar invariance stage were also compared with the fit indices obtained from the structural model. It was determined that the ∆RMSEA value was ≥.030 only in the educational and sociological sub-dimensions, but the ∆CFI (∆CFI ≤ .010) and ∆SRMR values (∆SRMR ≤.030) were below the expected cut-off value. The fact that the difference in more than one fit index was below the expected cut-off value indicates that scalar invariance was satisfied in these two sub-dimensions. The analysis was terminated since scalar invariance could not be provided in other sub-dimensions. Upon evaluating strict invariance for the educational and sociological sub-dimensions in which scalar invariance was satisfied, it was revealed that more than one fit index did not provide the expected difference, and strict invariance could not be met.

The results of measurement invariance for the six sub-dimensions of LS according to marital status are presented in Table 6. Upon examining the structural invariance, it was found that the fit indices of *χ*^2^/*df* and RMSEA values in all sub-dimensions, except for the relaxation sub-dimension, were above expectations. In the aesthetic and sociological sub-dimensions, it was observed that the NNFI value was close to the acceptable fit index, although it was slightly lower. However, other fit indices (SRMR, NFI, and CFI) indicated a good and perfect fit. The fit indices (NFI, NNFI, CFI, and SRMR) for the other sub-dimensions (Psychological, Educational, Physical, and Relaxation) also indicated a good and perfect fit, which can be interpreted as the fact that structural invariance was satisfied in all sub-dimensions. By achieving the structural invariance, the conditions of metric invariance, the second stage, were evaluated. It was observed that the ∆RMSEA value for all sub-dimensions was ≥ .015, but the ∆SRMR value was ≤ .030 and the ∆CFI value was ≤ .010, and metric invariance was satisfied with more than one fit index being in the expected range. Upon examining scalar invariance, which is the next stage after metric invariance, the values were calculated as ∆CFI ≥ .010, ∆RMSEA ≥. 015, and ∆SRMR ≥ .030 in almost all other sub-dimensions, except for the Physical satisfaction sub-dimension. This indicated that only metric invariance was satisfied in all sub-dimensions, except for the Physical satisfaction sub-dimension. When the scalar invariance, the next stage, was examined, the values were ∆RMSEA ≥. 015, ∆SRMR ≤ .030, and ∆CFI ≤ .010 for the Physical satisfaction sub-dimension. This indicated that scalar invariance was achieved. Upon examining strict invariance for the Physical satisfaction sub-dimension, the values were found to be ∆RMSEA ≥. 015, ∆SRMR ≥ .030, and ∆CFI ≥ .010, and all fit indices were above the expected cut-off value. This indicated that strict invariance was not satisfied for the Physical satisfaction sub-dimension.

**Table 6:** Fit statistics of measurement invariance stages by marital status

	Marital status	*χ*^*2*^	*df*	*χ*^*2*^*/df*	NFI	NNFI	CFI	SRMR	RMSEA (90% CI)	∆CFI	∆SRMR	∆RMSEA	Decision
Psychological	Structural	77.44	4	19.36	.98	.95	.98	.024	.125(.10–.15)	–	–	–	H_0_ Accept
	Metric	92.66	8	11.58	.98	.97	.98	.050	.095(.078–.11)	0	.026	−.030	H_0_ Accept
	Scalar	143.63	15	9.57	.97	.98	.97	.065	.086(.073–.099)	−.01	.041	−.039	H_0_ Reject
Educational	Structural	113.19	4	28.29	.98	.93	.98	.014	.153(.13–.18)	–	–	–	H_0_ Accept
	Metric	126.52	8	15.81	.97	.96	.97	.032	.112(.096–.13)	−.01	.018	−.041	H_0_ Accept
	Scalar	199.85	15	13.32	.96	.97	.96	.050	.103(.090–.12)	−.02	.036	−.050	H_0_ Reject
Social	Structural	198.36	4	49.59	.96	.88	.96	.031	.204(.18–.12)	–	–	–	H_0_ Accept
	Metric	241.56	8	30.19	.95	.93	.95	.061	.158(.14–.18)	−.01	.30	−.046	H_0_ Accept
	Scalar	304.26	15	20.28	.94	.95	.94	.064	.128(.12–.14)	−.02	.33	−.076	H_0_ Reject
Physical	Structural	114.66	4	28.66	.98	.94	.98	.034	.154(.13–.18)	–	–	–	H_0_ Accept
	Metric	117.26	8	14.65	.98	.97	.98	.035	.108(.091–.13)	0	.001	−.046	H_0_ Accept
	Scalar	202.99	15	30.19	.96	.97	.97	.059	.103(.091–.12)	−.01	.025	−.051	H_0_ Accept
	Strict	230.68	19	12.14	.96	.97	.96	.082	.098(0.86–.11)	−.02	.048	−.056	H_0_ Reject
Aesthetic	Structural	194.88	4	48.72	.95	.86	.95	.061	.202(.18–.23)	–	–	–	H_0_ Accept
	Metric	202.79	8	25.34	.95	.93	.95	.076	.144(.13–.16)	0	.015	−.058	H_0_ Accept
	Scalar	558.72	15	37.2	.85	.88	.85	.18	.181(.17–.19)	−.10	.119	−.021	H_0_ Reject
Relaxation	Structural	16.66	4	4.16	1.00	.99	1.00	.014	.052(.028–.079)	–	–	–	H_0_ Accept
	Metric	17.66	8	2.20	1.00	1.00	1.00	.027	.032(.011–.053)	0	.013	−.020	H_0_ Accept
	Scalar	173.41	15	11.5	.97	.98	.97	.094	.095(.083–.11)	−.03	.080	.043	H_0_ Reject

Table 7 contains the results of measurement invariance for the six sub-dimensions of LS according to age. It was observed that the fit indices of *χ*^2^/*df* and RMSEA values in all sub-dimensions, except for the relaxation sub-dimension, were above the acceptable ranges. In the aesthetic and sociological sub-dimensions, the NNFI value was found to be close to the acceptable fit criteria. All other fit indices (SRMR, NFI, and CFI) in these two sub-dimensions indicated a good and perfect fit. The fit indices (NFI, NNFI, CFI, and SRMR) for the other sub-dimensions (Psychological, Educational, Physical, and Relaxation) also showed a good and perfect fit. This can be interpreted as the fact that structural invariance was satisfied. By providing structural invariance, the conditions of metric invariance, the second stage, were evaluated. When the results on metric invariance for the psychological, sociological, physical, and aesthetic sub-dimensions were examined, the ∆RMSEA value was ≥. 015, but the ∆SRMR value was ≤ .030 and the ∆CFI value was ≤ .010. In the Educational and Relaxation sub-dimensions, whereas the ∆SRMR value was ≥.030, the ∆RMSEA value (≤.015) and the ∆CFI value (≤.010) were within the expected range. The fact that more than one fit index was in the expected value range can be interpreted that metric invariance was satisfied for all sub-dimensions. By providing metric invariance, the scalar invariance stage was evaluated, and the acquired fit indices were compared with the fit indices in structural invariance. While the differences between the fit indices for all sub-dimensions were above the cut-off value, the values were found to be ∆CFI ≥ .010, ∆RMSEA ≥.015, and ∆SRMR ≥.030, and scalar invariance was not satisfied in any of the sub-dimensions.

**Table 7:** Fit statistics of measurement invariance stages by age

	Age	*χ*^*2*^	*df*	*χ*^*2*^*/df*	NFI	NNFI	CFI	SRMR	RMSEA (90% CI)	∆CFI	∆SRMR	∆RMSEA	Decision
Psychological	Structural	76.54	8	9.56	.98	.95	.98	.025	.121(.097–.15)	–	–	–	H_0_ Accept
	Metric	103.54	20	5.17	.98	.98	.98	.055	.084(.069–.10)	0	.030	−.037	H_0_ Accept
	Scalar	224.60	35	6.41	.95	.97	.96	.066	.96(.084–.11)	−0.2	.041	−.839	H_0_ Reject
Educational	Structural	109.60	8	13.7	.98	.93	.98	.011	.147(.12–.17)	–	–	–	H_0_ Accept
	Metric	134.55	20	6.72	.97	.97	.97	.077	.134(.11–.15)	−0.1	.066	−.013	H_0_ Accept
	Scalar	234.64	35	6.70	.95	.97	.96	.087	.099(.087–.11)	−0.2	.076	−.048	H_0_ Reject
Social	Structural	192.85	8	24.1	.96	.89	.96	.029	.199(.17–.22)	–	–	–	H_0_ Accept
	Metric	253.43	20	12.6	.95	.94	.95	.056	.141(.13–.16)	−0.1	.027	−.058	H_0_ Accept
	Scalar	328.13	35	9.37	.94	.96	.94	.087	.120(.11–.13)	−0.2	.058	−.079	H_0_ Reject
Physical	Structural	126.73	8	24.1	.98	.93	.98	.038	.159(.14–.18)				H_0_ Accept
	Metric	155.41	20	7.77	.97	.97	.97	.062	.108(.092–.12)	−0.1	.024	−.051	H_0_ Accept
	Scalar	273.50	35		.95	.97	.95	.13	.108(.096–.12)	−0.2	.092	−.051	H_0_ Reject
Aesthetic	Structural	176.85	8	22.1	.96	.87	.96	.075	.190(.17–.21)	–	–	–	H_0_ Accept
	Metric	210.03	20	10.5	.95	.94	.95	.10	.127(.11–.14)	−0.1	.025	−.063	H_0_ Accept
	Scalar	669.63	35	19.1	.83	.89	.84	.20	.176.(.18–.19)	−.11	.125	−.014	H_0_ Reject
Relaxation	Structural	22.95	8	2.86	1.00	.99	1.00	.016	.057(.030–.084)	–	–	–	H_0_ Accept
	Metric	42.58	20	2.12	.99	1.00	1.00	.096	.044(.025–.062)	0	.080	−.013	H_0_ Accept
	Scalar	236.55	35	6.75	.96	.98	.97	.13	.099(.087–.11)	−0.3	.114	.042	H_0_ Reject

## Discussion

Upon reviewing studies in social sciences, it is seen that some studies are about the comparisons between groups. However, the current studies are based on the premise that the measurement tool used to reveal the difference between groups measures the same feature in those subgroups, and it is possible to talk about the accuracy of the comparisons made by meeting this premise. Otherwise, the significance of the comparisons between groups is controversial.

Numerous studies on LS addressed intergroup differences. According to Park and Chon (2010), it is very important to develop different scales for leisure activities and validate these scales. However, it was indicated that many researchers did not investigate the invariance of measurement tools according to demographic variables (e.g., gender, age, marital status), and it was important for researchers to verify these scales before using the scales developed in the field (Ahn & Song, 2021). In this respect, the present study aimed to test whether the differences obtained according to gender, marital status, and age were caused by the measurement tool itself using LSS, and it was found that the differences to be obtained did not originate from the bias of the measurement tool.

Considering the research results, it was determined that structural invariance was satisfied according to gender, marital status, and age in all sub-dimensions of LS (Psychological, Educational, sociological, physical, aesthetic, and relaxation). Providing structural invariance revealed that the structure of LSS was the same according to gender, marital status, and age, and the conceptual perspectives of individuals were invariant while answering the items related to all sub-dimensions of this scale (Vandenberg & Lance, 2000).

Upon evaluating metric invariance for LSS, it was determined that metric invariance was satisfied in all sub-dimensions according to gender, marital status, and age. Metric invariance is one of the important stages of measurement invariance. Providing metric invariance indicates that similar responses were given to these items while responding to the scale items of all subgroups. In other words, the measured characteristics of the scale in the subgroups are similar. Providing metric invariance reveals that the results obtained from the comparisons made in the subgroups do not originate from the measurement tool; thus, the comparisons made are significant (Byrne & Watkins, 2003).

The results of scalar invariance showed that scalar invariance was satisfied in the educational and sociological sub-dimensions according to gender and only in the physical satisfaction sub-dimension according to marital status. Scalar invariance reveals that the factor loadings of the items are comparable in the scales it is provided. In cases where the educational and sociological satisfaction sub-dimensions by gender and physical satisfaction by marital status are compared with scalar invariance, the constant in the regression equation is the same, and the mean scores of latent factor scores can be compared according to these variables. Scalar invariance refers to the fact that there is no bias between the groups on an item basis. Furthermore, the study results showed that strict invariance was not satisfied according to gender, marital status, and age in the sub-dimensions where scalar invariance was satisfied. The cases where strict invariance is not satisfied indicate that the error terms of the items may change according to the variable examined. In other words, the specific variances of the items cannot be compared. Researchers working in different disciplines argue that error variance invariance (strict invariance) is not required for item analyses (Wang & Wang, 2019). However, if there is a difference between the reliability of the items between the groups, error variance invariance is important. The reason for this is explained as follows: Considering that factor variances between groups are invariant (Vandenberg & Lance, 2000), the invariance of error variances is considered the invariance of item reliability between the groups compared (Schmitt et al., 1984). Four hundred forty-eight adult men and women from Seoul, Gyeonggi, Chungcheong, and Gangwon-do, Korea, participated in a study that tested the measurement invariance of LSS developed on Korean adult individuals (Ahn & Song, 2021). The study results revealed that men and women had a similar conceptual point of view while responding to the questions of LSS, their answers to the items were similar, and the constant in the regression equation for the items was the same for men and women. In other words, the structural, metric, and scalar invariance of this measurement tool by gender was satisfied but strict invariance was not (Ahn & Song, 2021). The fact that the structural, metric, and scalar invariance of LSS according to the gender variable in the context of the feature being measured was satisfied but strict invariance was not is very important because it is similar to our study results. The findings allowed the results obtained from the differences between the groups regarding LS to be reported more accurately and contribute to the field both academically and practically.

## Limitations and future research

Our study is limited to adult individuals who were registered to sports centers in Antalya province and regularly engaged in physical activity. Therefore, it is important to re-evaluate invariance by gender, age, and marital status with individuals living in different cities since the study results cannot be generalized across the country. Furthermore, in this study, we tested measurement invariance by gender, marital status, and age variables, which are mostly compared in the literature. The fact that researchers working on this subject will conduct studies on measurement invariance with other variables, such as leisure type, frequency of leisure participation, education, and income level, in the future may provide more comprehensive results for the construct validity of the measurement tool. However, the importance of measurement invariance studies in the field of leisure has been recognized by researchers and started to be reported in some measurement tools (Cho et al., 2020; Köse et al., 2021; Köse et al., 2022; Liu et al., 2014). It will be important for researchers to provide construct validity for other measurement tools in the field of leisure such as leisure crafting, involvement, attitude, boredom, serious leisure, and more for the validity of the research findings to be obtained from intergroup comparisons.

In some studies, in which measurement invariance is performed, the invariance of the items (components) of the measurement tool cannot be met (Cheung, 2008; Vandenberg & Lance, 2000). In this case, it may be recommended to reevaluate and reorganize the items in order to eliminate the biases regarding the items in the measurement tool, and then perform the analysis again according to the socio-demographic variable tested. If invariance is not satisfied even in this case, partial invariance studies can be conducted in the groups where invariance is tested.

## Conclusion

This study provides important evidence regarding the structural/construct validity of the LLS. The measurement invariance of leisure motivation according to gender, marital status, and age (configural, weak, strong, and strict) was evaluated. The tool measures the same psychological structure according to gender, marital status (configural) and age, and all subgroups respond to the scale items the same way (metric). This makes it possible to compare the scores obtained from the measurement tool on a group basis. Scalar invariance was achieved in the sub-dimensions of education and social satisfaction according to gender and in the sub-dimension of physical satisfaction according to marital status. In addition, the results consistently showed that strict invariance is not satisfied for any sub-group and sub-dimension.

## Data Availability

The datasets generated during and/or analysed during the current study are available from the corresponding author on reasonable request.

## Abbreviations

LS: Leisure Satisfaction
LSS: Leisure Satisfaction Scale
MG-CFA: Multiple-Group Confirmatory Factor Analysis
CFA: Confirmatory Factor Analysis
VIF: Variance Inflation Factor
CI: Condition Index
χ2: Chi-square
df: Degree of freedom
CFI: Comparative Fix Index
NFI: Normed Fit Index
NNFI: Non-Normed Fit Index
RMSEA: Root Mean Square Error of Approximation
SRMR: Standardized Root Mean Square Residual
